# Thermocatalytic Degradation of Gaseous Formaldehyde Using Transition Metal‐Based Catalysts

**DOI:** 10.1002/advs.202300079

**Published:** 2023-04-28

**Authors:** Yongbiao Hua, Younes Ahmadi, Ki‐Hyun Kim

**Affiliations:** ^1^ Department of Civil and Environmental Engineering Hanyang University 222 Wangsimni‐Ro Seoul 04763 South Korea

**Keywords:** formaldehyde, kinetic reaction rate, reaction mechanism, thermocatalysis, transition metal‐based catalysts

## Abstract

Formaldehyde (HCHO: FA) is one of the most abundant but hazardous gaseous pollutants. Transition metal oxide (TMO)‐based thermocatalysts have gained much attention in its removal due to their excellent thermal stability and cost‐effectiveness. Herein, a comprehensive review is offered to highlight the current progress in TMO‐based thermocatalysts (e.g., manganese, cerium, cobalt, and their composites) in association with the strategies established for catalytic removal of FA. Efforts are hence made to describe the interactive role of key factors (e.g., exposed crystal facets, alkali metal/nitrogen modification, type of precursors, and alkali/acid treatment) governing the catalytic activity of TMO‐based thermocatalysts against FA. Their performance has been evaluated further between two distinctive operation conditions (i.e., low versus high temperature) based on computational metrics such as reaction rate. Accordingly, the superiority of TMO‐based composite catalysts over mono‐ and bi‐metallic TMO catalysts is evident to reflect the abundant surface oxygen vacancies and enhanced FA adsorptivity of the former group. Finally, the present challenges and future prospects for TMO‐based catalysts are discussed with respect to the catalytic oxidation of FA. This review is expected to offer valuable information to design and build high performance catalysts for the efficient degradation of volatile organic compounds.

## Graphical abstract

Formaldehyde is one of the most abundant but hazardous gaseous pollutants. Transition metal oxide (TMO)‐based thermocatalysts have gained much attention in its removal due to their excellent thermal stability and cost‐effectiveness. Herein, the current progress in TMO‐based thermocatalysts is reviewed in association with their preparation/modification strategies, performance evaluation, and the interactive role of key factors governing their catalytic activities.

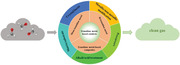

## 1 Introduction

Formaldehyde (FA) is represented as one of the simplest forms of volatile organic compounds (VOCs). FA is often regarded as the priority target for treatment because of its ubiquity, abundance, and negative effects on human health.^[^ ^1^ ^]^ Sources of indoor FA are diverse to include cooking, smoking, building decoration, and furnishing materials (like glue, varnishes, plastic castings, rubber, and wooden furniture).^[^ ^2^ ^]^ In light of the adverse health effects of FA, the World Health Organization has set its short‐term (30 min) exposure limit in indoor environment at 0.1 mg m^−3^.^[^ ^3^ ^]^ There is even more strict technical standard of 0.03 mg m^−3^ for the emission control in interior decoration systems such as residential buildings in China.^[^ ^4^ ^]^ As people are spending more of their time indoors, it is critical to control FA levels in indoor air to avoid their adverse effects on human health.

Recently, the thermocatalytic degradation approach is recognized as an effective option to achieve the complete decomposition of gaseous FA into harmless products (e.g., H_2_O and CO_2_) under atmospheric pressure with heat energy.^[^ ^5^ ^]^ As thermocatalysis can proceed in the absence of a light source, it can be regarded as a superior option over photocatalytic degradation method.^[^ ^6^ ^]^ Generally, the thermocatalytic degradation of gaseous FA has been achieved using noble (e.g., platinum, gold, palladium, rhodium, and silver) and/or transition metal (TM) (e.g., manganese) based materials.^[^ ^7^ ^]^ However, their real‐world applications are often restricted by the combined effects of both low abundance of noble metals and their high costs.^[^ ^8^ ^]^ As a result, TM‐based catalysts have gained interest as alternative options due to their high stability, good catalytic activity, abundant resource, and cost‐effectiveness.^[^ ^3^ ^]^ TM catalysts can offer unpaired d electrons or empty d orbitals to efficiently attract target molecules during the catalysis process.^[^ ^9^ ^]^ As such, the catalytic destruction of pollutants over these TMs can be promoted by forming chemical bonds and/or by lowering the activation energy of the reaction.^[^ ^9^ ^]^ Moreover, the catalytic activities of TM‐based composites were also reported to be higher than those of their pure forms such as Co_3_O_4_, CeO_2_, and MnO_2_.^[^ ^10^ ^]^ Such enhancement was mainly due to the improved oxidation capacity of the composites with higher FA adsorptivity and/or oxygen vacancies.^[^ ^11^ ^]^

The objectives of this review are to highlight the recent progress in the development of TM‐based thermocatalysts (e.g., manganese, cerium, cobalt, and their composites) for the degradation of gaseous FA. The discussion has been extended to cover their structural activity relationship and their FA degradation mechanism. Effective strategies (e.g., exposed specific crystal faces, metallic/non‐metallic modification, and alkali/acid treatment) developed for improving their catalytic activities are also discussed. Moreover, the FA degradation efficiencies of TM‐based thermocatalysts are also evaluated in terms of the key performance metrics (e.g., T_90_ and kinetic reaction rate) to properly evaluate their potential for real‐world applications. Finally, the current challenges and outlook of the research are also discussed. The current review is expected to deliver salient information for the construction of highly active TMO‐based thermocatalysts for the effective removal of gaseous FA.

## 2 Reaction Mechanism on TM‐Based Thermocatalysts

A better knowledge of the catalytic oxidation mechanism of FA by TM‐based thermocatalysts can help in the design of highly active, stabile, and efficient thermocatalysts. As thermocatalysts are exposed to the external heat energy, electrons (e^−^) from valence band are excited to the conduction band to leave positive holes (h^+^) in the valence band even at low temperature (e.g., room temperature).^[^ ^12^ ^]^ The concentration of e^−^/h^+^ in thermocatalysts generally increases at elevated temperature (e.g., 50 °C).^[^ ^12^ ^]^ The thermally generated charge carriers further move to the surface of the catalyst to react with adsorbed oxygen molecules and water for the generation of surface adsorbed oxygen species such as superoxide anions (O_2_^−^, e^−^ + O_2_ → O_2_^−^) and hydroxyl radicals (^●^OH, h^+^ + H_2_O → ^●^OH). Further, water can also be converted into O_2_ and ^●^OH with the assistance of O_2_^−^.^[^ ^12b^ ^]^ The target pollutants can thus be catalytically oxidized to CO_2_ and H_2_O by these surface adsorbed oxygen species.^[^ ^12^, ^13^ ^]^ The production of surface adsorbed oxygen species is also affected by the amount of oxygen vacancies (OVs) of the thermocatalysts (e.g., Co_3_O_4_ and MnO_2_).^[^ ^14^ ^]^ The surface oxygen vacancies possess localized electrons that can easily charge adsorbed oxygen (^●^O^2−^, O_2_^2−^, or O_4_^2−^) via the molecular oxygen activation channel.^[^ ^15^ ^]^ Hence, the O_2_ molecules can accept delocalized electrons from oxygen vacancies (i.e., Lewis bases) to be converted into active species.^[^ ^4^, ^14^ ^]^

The creation of surface defects (e.g., through doping and pyrolysis) over the thermocatalysts is also recognized as an effective way to achieve abundant oxygen vacancies.^[^ ^16^ ^]^ In addition, higher concentrations of oxygen vacancies in the thermocatalysts can narrow the band gap to enhance the catalytic activity against VOC degradation.^[^ ^17^ ^]^ The lattice oxygen species were also found to enhance the formation of surface adsorbed oxygen species through its complex interaction with oxygen vacancy and molecularly oxygen.^[^ ^18^ ^]^ As such, the FA catalytic activity of catalysts was enhanced.^[^ ^18^ ^]^ For instance, the FA catalytic activity for four MnO_2_ catalysts (i.e., *α*, *β*, *γ*, and *δ*‐MnO_2_) was in line with their amount of lattice oxygen with the following relative order: *δ*‐MnO_2_ > *α*‐MnO_2_ > *β*‐MnO_2_ > *γ*‐MnO_2_.^[^ ^19^ ^]^

**Figure 1:**
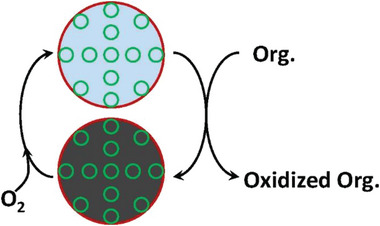
Schematic of the Mars‐van Krevelen mechanism in the thermocatalytic degradation of organic pollutants (Org.), Reproduced with permission.^[^ ^21b^ ^]^ Copyright 2019, Elsevier.

**Figure 2:**
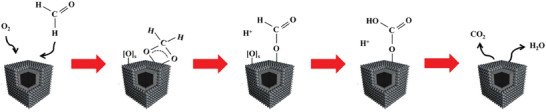
Illustration of FA oxidation over cobalt‐manganese oxides catalysts, Reproduced with permission.^[^ ^22^ ^]^ Copyright 2022, Elsevier.

The thermocatalytic oxidation of FA over transition‐metal based catalysts (e.g., manganese and cobalt oxides) was found to follow a Mars‐van Krevelen mechanism in which OVs can play a vital role during the degradation process.^[^ ^4^, ^7^, ^16^, ^20^ ^]^ In such a mechanism, pollutants are initially oxidized by the surface adsorbed oxygen species to lead to the reduction of metal sites (**Figure** **1**). Then, the reduced meal centers are re‐oxidized by O_2_.^[^ ^21^ ^]^ A detailed multi‐step catalytic process for oxidation of FA through Mars‐van Krevelen mechanism (e.g., over TM cobalt‐manganese oxides) has been proposed (**Figure** **2**).^[^ ^22^ ^]^ First, FA and oxygen molecules are adsorbed on the catalysts surface and active sites (e.g., oxygen vacancies), respectively. The oxygen molecules are dissociated and activated into surface adsorbed oxygen species (e.g., O_2_^−^ and O^−^) by oxygen vacancies at low/high temperature (O_2_ + OVs → O_2_^−^, O^−^).^[^ ^23^ ^]^ Then, the adsorbed FA molecules rapidly react with surface adsorbed oxygen species to be converted into dioxymethylene intermediates and further into formate species so as to form hydrocarbonates. These hydrocarbonate species are to be ultimately oxidized into H_2_O and CO_2_.^[^ ^22^ ^]^ Similar oxidation pathways were also found when pure/surface defected manganese and cobalt catalyst were used for the thermal oxidation of FA.^[^ ^4^, ^16^, ^20^, ^24^ ^]^ In addition, thermocatalytic degradation of FA over noble metal catalysts (e.g., Pt/TiO_2_) can also be explained by a similar Mars‐van Krevelen mechanism.^[^ ^25^ ^]^ More specifically, the FA molecules were adsorbed on the surface of catalysts and then directly oxidized into formate species by surface adsorbed oxygen species. The formate species were subsequently decomposed into H_2_O and CO which was further oxidized into CO_2_.^[^ ^25a^ ^]^ Interestingly, as the existence of strong metal‐support interactions in Pt/TiO_2_ catalysts can cause the partial reduction of the Ti^4+^ species into Ti^3+^, it can favorably enhance the FA catalytic activity through the generation of OVs.^[^ ^25a^ ^]^ If the surface of the thermocatalysts possesses abundant surface hydroxyl (—OH) groups, the adsorption of FA on the thermocatalyst's surface can be enhanced to induce its subsequent degradation with the assistance of surface —OH.^[^ ^14b^ ^]^ In addition, the formate intermediate species might be directly oxidized to CO_2_ and H_2_O by surface —OH groups (HCOO^−^ + —OH → CO_2_ + H_2_O) during the FA oxidation process.^[^ ^26^ ^]^ Therefore, the surface —OH groups are considered the main factor for determining the rate of FA oxidation.^[^ ^26a^ ^]^

## 3 TM‐Based Thermocatalysts

The TM‐based thermocatalysts can be classified based on their composition (such as mono‐TM oxides (mono‐TMO), bi‐TMO, and composite transition metal oxide (TMO)‐based thermocatalysts) with regard to FA oxidation.

### 3.1 Mono‐TMO Based Thermocatalysts

A plethora of mono TMO‐based thermocatalysts (e.g., manganese, cobalt, chromium, and cerium oxides) have been developed for the degradation of FA.^[^ ^20^, ^27^ ^]^ Such thermocatalysts possess favorable pore structure, high surface area, good catalytic activity, high stability, and low cost, and they can be used for environmental remediation,^[^ ^3^, ^5^, ^27^, ^28^ ^]^ Many approaches have been proposed for developing TM‐based catalysts such as sol‐gel, hydrothermal, precipitation, and template methods.^[^ ^24^, ^29^ ^]^ As the preparation of TM‐based thermocatalysts has been discussed previously, interested readers may refer to the synthesis methods described elsewhere.^[^ ^5^, ^7^, ^30^ ^]^

**Figure 3:**
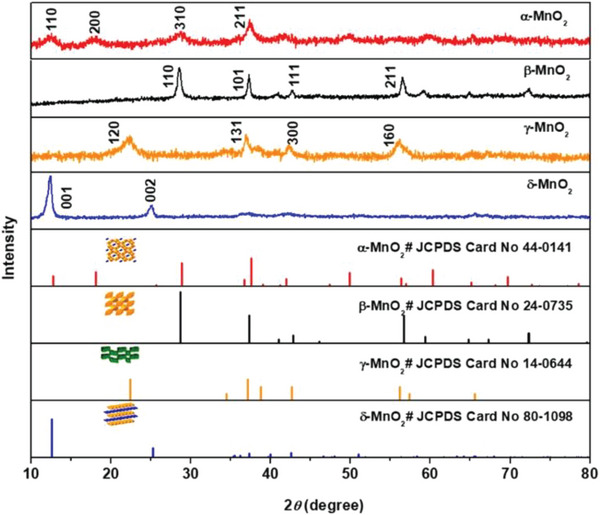
The XRD patterns for different forms of manganese oxide: *α*‐MnO_2_ (red line), *β*‐MnO_2_ (black line), *γ*‐MnO_2_ (orange line), and *δ*‐MnO_2_ (blue line), Reproduced with permission.^[^ ^31^ ^]^ Copyright 2020, American Chemical Society (ACS).

Monometallic TMO‐based thermocatalysts can exist in several crystallographic structures.^[^ ^19^ ^]^ In particular, manganese oxide can exist in many different forms of crystals (e.g., *α*‐MnO_2_, *β*‐MnO_2_, *γ*‐MnO_2_, and *δ*‐MnO_2_), which consist of [MnO_6_] octahedra to share corners and edges in their structure (**Figure** **3**).^[^ ^31^ ^]^ The presence of variable chemical valences and defects of manganese oxides will help increase the mobility of surface oxygen to boost their FA degradation ability with the enhanced oxygen storage capacity.^[^ ^28^, ^32^ ^]^ For instance, the birnessite MnO_2_, with edge‐sharing octahedral MnO_6_ layers, was reported to exhibit 100% FA degradation capacity at room temperature.^[^ ^23c^ ^]^ Such birnessite structures can provide higher water content (like interlayers and adsorbed water) to amplify the thermocatalytic activity.^[^ ^23c^ ^]^ Water molecules can also help generate the consumed surface —OH groups (during the FA oxidation) through the reaction with surface active oxygen species (O_2_^−^, O^−^ + H_2_O → 2‐OH).^[^ ^23c^ ^]^

**Figure 4:**
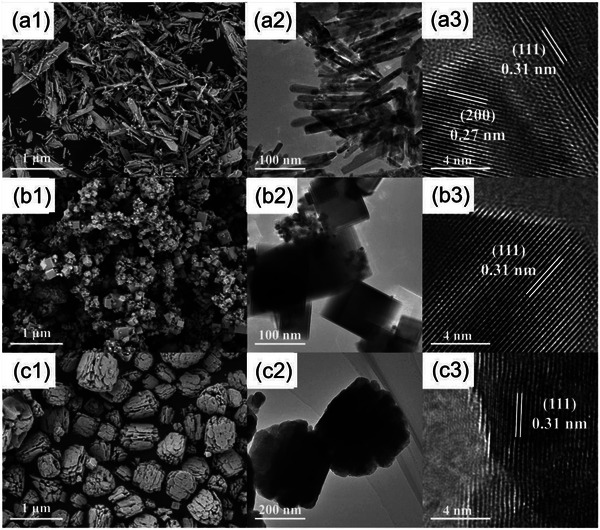
The SEM, TEM, and HRTEM images of CeO_2_: a1–a3) CeO_2_ spherical‐like aggregate of nanoplates, b1–b3) CeO_2_ nanorods, and c1–c3) CeO_2_ nanocubes, Reproduced with permission.^[^ ^34^ ^]^ Copyright 2022, Elsevier.

The monometallic TMO‐based thermocatalysts can also be designed into various morphologies (e.g., sheet, cube, and rod).^[^ ^20^, ^28^, ^29^, ^33^ ^]^ For instance, the CeO_2_ spherical‐like aggregate of nanoplates exhibited excellent FA oxidation activity (e.g., relative to CeO_2_ nanorods and nanocubes) with the aid of the abundant surface hydroxyl and oxygen groups (**Figure** **4**): It was reported to achieve a maximum conversion of 87% against 500 ppm FA at 120 °C with a GHSV of 10 L g^−1^ h^−1^.^[^ ^34^ ^]^ A stable fluorite structure of CeO_2_ (consisting Ce^4+^ and Ce^3+^ ions) with a 2D sheet‐like morphology and high concentration of OVs was also observed to completely degrade 50 ppm FA at 310 °C.^[^ ^16a^ ^]^ In another study, a 3D MnO_2_ structure with an interconnected network structure was developed through a freeze‐drying method.^[^ ^33b^ ^]^ The 3D monolith network facilitated the diffusion of reactants onto the active sites of the catalyst. As such, the 3D MnO_2_ was able to fully destruct FA (100 ppm) at 80 °C under a gas hourly space velocity (GHSV) of 180 L g^−1^ h^−1^.^[^ ^33b^ ^]^ In addition, Co_3_O_4_ nano‐rods were built to have a large surface area and a high content of surface Co^3+^. Such characteristics offered large numbers of oxygen anionic sites to promote the adsorption of H_2_O molecules. The adsorbed water molecules can further be dissociated to form ^●^OH active species for the oxidation of FA.^[^ ^28b^ ^]^

Despite the extensive research on monometallic TMO‐based thermocatalysts (**Table** **1**), they suffer from several disadvantages (e.g., poor ability of O_2_ activation and limited oxygen vacancies) that affect their FA degradation efficiency.^[^ ^3^, ^4^ ^]^ For example, high temperature (>90 °C) (e.g., urchin‐like MnO_2_, 2D‐Co_3_O_4_, and CeO_2_ nanorod) is often required to achieve complete FA oxidation (Table 1).^[^ ^29^, ^34^, ^35^ ^]^ Hence, researchers have produced thermocatalysts that are more effective than monometallic TM‐based thermocatalysts by introducing impurities and other enhancing agents as discussed below.

**Table 1:** Summary of transition metal oxide thermocatalysts for catalytic degradation of FA under low temperature conditions

Order	Catalyst	Preparation method	Catalyst mass [mg]	Reactant mixture	Pollutant concentration [ppm]	Flow rate [mL min^−1^]	Flow rate [mol s^−1^]	Space velocity	T_90_ [°C]	Conversion [%] at RT	*r* [mmol mg_cat_^−1^ h^−1^]	BET [m^2^ g^−1^]	Reference
[A] Mono transition metal oxide‐based thermocatalysts													
1	Birnessite MnO_2_	Redox reaction	50	200 ppm FA + air + 65%RH	10	300	2.04E‐09	180 000 h^−1^	RT	85	1.25E‐02	21	[23c]
2	Co_3_O_4_ nanobelt	Topochemical transformation method	50	260 ppm FA + 20%RH	260	NA	NA	NA	RT	100	NA	61	[14a]
3	Co_3_O_4_ nanoplate	Hydrothermal method	50	260 ppm FA + 20%RH	260	NA	NA	NA	RT	52	NA	21	[14a]
4	Co_3_O_4_ nanosheet	Hydrothermal method	50	260 ppm FA + 20%RH	260	NA	NA	NA	RT	5.7	NA	39	[14a]
[B] Transition metal oxide‐based composites thermocatalysts													
5	Carbon sphere@MnO	Ambient reaction	100	60 ppm FA + 21%O2 + N2 + O3 + 50%RH	60	100	4.09E‐09	60 000 mL g^−1^ h^−1^	RT	100	1.47E‐02	102.1	[61]
6	Carbon sphere/MnO_2_	Impregnated method	100	2 ppm FA + Air	2	1000	1.36E‐09	80 000 h^−1^	RT	100	4.91E‐03	1212.21	[43]
7	Birnessite/granular activated carbon	In situ reduction method	500	0.4 ppm FA + air + 45%RH	0	1000	2.73E‐10	NA	RT	74	1.45E‐04	NA	[45]
8	MnO*_x_*/activated carbon	In situ synthesis method	NA	10 ppm FA + air + 50%RH	10	1000	6.82E‐09	65 000 h^−1^	RT	100	NA	411.9	[62]
9	*δ*‐MnO_2_/activated carbon fiber	Co‐precipitation method	200	15 ppm FA	15	200	2.04E‐09	60 000 mL g^−1^ h^−1^	RT	25	9.20E‐04	679.12	[63]
10	Ammonia modified *δ*‐MnO*_x_*/activated carbon	Impregnated method	100	100 ppm FA + 21%O_2_ + N_2_ + 20 or 50% RH	100	100	6.82E‐09	60 000 mL g^−1^ h^−1^	RT	97.5	2.39E‐02	714.81	[23b]
11	*δ*‐MnO_2_/palygorskite	Impregnated method	100	1 ppm FA + air	1	900	6.13E‐10	150 000 h^−1^	RT	90	1.99E‐03	73.2	[64]
12	MnO_2_/PET	Impregnated method	NA	200 ppm FA	200	NA	NA	NA	RT	85	NA	NA	[65]
13	MnO*_x_*/PET	In situ synthesis method	500	1 ppm FA + air	1	1000	3.41E‐10	17 000 h^−1^	RT	90	2.21E‐04	NA	[41b]
14	Cotton/pDA/MnO_2_	In situ synthesis method	NA	22.7 ppm FA	23	NA	NA	NA	RT	100	NA	NA	[66]
15	MnO_2_‐Nonwoven	Calendaring method	NA	0.9 ppm FA + air	0.9	NA	NA	NA	RT	90	NA	NA	[67]
16	MnO*_x_*/PMMA/SSM	Electrostatic spinning and hydrolysis method	1000	11 ppm FA + 45%RH	11	NA	NA	NA	RT	100	NA	107.68	[68]
17	MnO_2_/PEG	Precipitation method	100	5 ppm FA + 60% RH	5	400	1.36E‐09	240 000 mL g^−1^ h^−1^	RT	96.8	4.75E‐03	31.2	[44a]
18	Carbon/Co_3_O_4_	Sol‐gel method	300	1 ppm FA	1	500	3.41E‐10	100 000 mL g^−1^ h^−1^	RT	90	3.68E‐04	186.87	[23a]

### 3.2 Bi‐TMO‐Based Thermocatalysts

Bi‐TMO‐based catalysts have been reported to exhibit superior catalytic activities for FA compared to their mono metallic counterparts.^[^ ^13^, ^36^ ^]^ Bi‐TMO‐based catalysts are generally fabricated through a sol‐gel method, a template method, precipitation, and a hydrothermal method.^[^ ^13^, ^36^, ^37^ ^]^ The improved FA oxidation capabilities of bi‐TMO‐based thermocatalysts (e.g., MnO*_x_*‐CeO_2_, Co_3_O_4_‐CeO_2_, MnO_2_‐Fe_2_O_3_, CuO‐MnO_2_, and Co_3_O_4_‐ZrO_2_) is due to their high surface oxygen mobility and OVs, which help promote the transportation of charges during redox cycles.^[^ ^3^, ^36^ ^]^ In this regard, mono‐TMO‐based thermocatalysts such as cerium oxides are frequently bound with other TMOs (e.g., cobalt oxides).^[^ ^37a^ ^]^ In such a case, the combination of two metal oxides helps synergize the overall thermocatalytic degradation capabilities of FA. For instance, cerium oxides in CeO_2_‐Co_3_O_4_ thermocatalysts offer high oxygen storage capacity, good redox performance, and high lattice oxygen activity, while Co_3_O_4_ provides strong oxidation activity with good electron transfer properties^[^ ^4^ ^]^ (Table 1). Similarly, the variable valence, easy defect formation, and high activity of manganese oxides give them high potential for bi‐TM‐based thermocatalysts.^[^ ^4^, ^38^ ^]^ For example, the complete oxidation of FA at lower temperature (100 °C) can be achieved by the synergy between manganese and cobalt oxides in Co*_x_* Mn_3‐_ *_x_* O_4_ catalysts compared to each of their pristine forms, that is, MnO*_x_* (170 °C) or CoO*_x_* (180 °C).^[^ ^38^ ^]^ The synergy was obtained through a series of redox cycles including Mn^4+^/Mn^3+^ and Co^3+^/Co^2+^ involving the activation of oxygen molecule by Co and its transfer to Mn.^[^ ^38^ ^]^ Generally, the FA oxidation reaction over bi‐TMO‐based thermocatalyst (e.g., Co*_x_* Mn_3‐_ *_x_* O_4_) follows the formate decomposition route (i.e., HCHO→ HCOO^−^→ CO→ CO_2_).^[^ ^38^ ^]^

**Figure 5:**
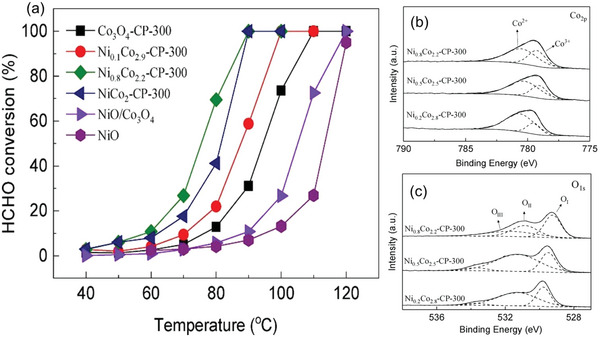
The formaldehyde oxidation using Ni*_x_* Co_3‐_ *_x_* O_4_ catalysts: a) Temperature dependence of formaldehyde conversion over Ni*_x_* Co_3‐_ *_x_* O_4_ catalysts with varying ratios of Ni/Co prepared by co‐precipitation method at 300 °C (Ni*_x_* C_3‐_ *_x_* O_4_‐CP‐300) and b,c) XPS spectra for Ni*_x_* C_3‐_ *_x_* O_4_‐CP‐300: Co_2p_ and O_1s_, Reproduced with permission.^[^ ^40^ ^]^ Copyright 2019, Elsevier.

The ratio of TMs in bi‐TMO‐based catalysts can significantly influence their catalytic activities against FA.^[^ ^13^, ^37^, ^39^ ^]^ For instance, Ni_0.8_Co_2.2_ oxides synthesized by co‐precipitation method at 300 °C (Ni_0.8_Co_2.2_‐CP‐300) had the best FA catalytic activity (complete removal of 100 ppm FA at 90 °C) among all Ni*_x_* Co_3‐_ *_x_*‐CP‐300 (*x* = 0–1) catalysts (**Figure** **5**a).^[^ ^40^ ^]^ The outstanding activity of Ni_0.8_Co_2.2_‐CP‐300 was ascribable to the surface oxidant Co^3+^ and abundant hydroxyl as evidenced in X‐ray photoelectron spectroscopy (XPS) spectrum (Figure 5b,c).^[^ ^40^ ^]^ As another example, the effect of Ce amount on the thermocatalytic performance of CeO_2_‐Co_3_O_4_ was investigated.^[^ ^37a^ ^]^ Accordingly, the best FA oxidation performance (100% of FA removal at 80 °C) was found for a Co/(Co + Ce) atomic ratio of 0.95. Moreover, according to O_2_‐temperature‐programmed desorption (TPD) analysis, the large desorption peak of O_2_ in CeO_2_‐Co_3_O_4_ (ratio of 0.95) thermocatalyst also indicated an increased O_2_ adsorption capacity, which helped produce surface active oxygen.^[^ ^37a^ ^]^ In contrast, the pure Co_3_O_4_ (containing no Ce) showed only 35% FA removal under the same temperature (80 °C). Such a difference could be attributed to the increase of surface adsorbed oxygen species as evidenced by H_2_‐ temperature programmed reduction analysis. However, at higher Co/(Co + Ce) atomic rations (>0.95) the phase separation resulted in lower FA catalytic activities for the catalyst.^[^ ^37a^ ^]^ Likewise, when the ratio of Co:Mn was 3:1, Mn*_x_* Co_3‐_ *_x_* O_4_ possessed the highest amount of surface oxygen and exhibited the best catalytic activity against FA relative to other Co:Mn ratios (e.g., 8:1, 2:1, and 1:1). As such, Mn*_x_* Co_3‐_ *_x_* O_4_ exhibited the best catalytic performance to achieve 100% oxidation of 80 ppm FA at a lower temperature (75 °C) than that of its pure counterparts (i.e., MnO_2_ (90 °C) and Co_3_O_4_ (105 °C)).^[^ ^39a^ ^]^

### 3.3 TMO‐Based Composite Thermocatalysts

Despite the good performance of pure TMO‐based thermocatalysts (e.g., mono‐ and bi‐TMO), their application in powder or particle forms often gives rise to dust contamination, which complicates the process.^[^ ^41^ ^]^ In addition, as other TMO forms (e.g., nanoparticles, nanorods, and nanoplates) tend to agglomerate, their catalytic performance can be degraded.^[^ ^42^ ^]^ Therefore, various nanomaterials (e.g., carbon spheres and polyester fiber) with advanced properties (e.g., high specific surface area and porosity) have been coupled with TMOs to fabricate TMO‐based composites.^[^ ^41^, ^43^ ^]^ In addition, the utilization of various nanomaterials as matrix/substrate has also been recognized as an effective route to increase the FA oxidation performance over TMO‐based catalysts by imparting abundant surface oxygen vacancies.^[^ ^10^, ^44^ ^]^

The TMO‐based composite catalysts are generally formulated through solution mixing, calcination, and in situ approaches.^[^ ^41^, ^43^, ^45^ ^]^ To prepare the MnO_2_ modified activated carbon (MnO_2_/AC) spheres, the AC spheres were impregnated with Mn(NO_3_)_2_·4H_2_O solution and then dried at 105 °C for 24 h. The final product was obtained through a calcination process at 300 °C for 3.5 h under a nitrogen atmosphere.^[^ ^43^ ^]^ For the in situ approach, the TMOs can be synthesized in the presence of the matrix for the generation of stable composites.^[^ ^41^ ^]^ The synthesis approach has also influenced the thermocatalytic properties of TMO‐based composites. For example, the crystallinity of Co_3_O_4_ was lowered after the in situ growth of Co_3_O_4_ nanowires on the Ni foam surface. This approach helped introduce more oxygen vacancies for oxidation of FA.^[^ ^10b^ ^]^ Accordingly, the reduced (r)‐Co_3_O_4_ NW@Ni foam composites possessed more active oxygen species with high mobility and reactivity with the aid of increased surface OVs. Such abundant surface OVs could lower O_2_ adsorption energy to make r‐Co_3_O_4_ NW@Ni foam composites easily adsorb and store more active oxygen species. As such, r‐Co_3_O_4_ NW@Ni foam composites exhibited the best FA oxidation performance with a much reduced T_10_ (i.e., temperature for 10% FA conversion) of 75 °C compared to the pristine Co_3_O_4_ (132 °C).^[^ ^10b^ ^]^

Matrices including carbonaceous materials (e.g., activated carbon fibers, granular activated carbon, and activated carbon spheres), polyester fibers, and cellulose fibers, generally show improved surface area and porosity in the synthesis of TMO‐based composite catalysts.^[^ ^41^, ^43^, ^45^, ^46^ ^]^ As an example, CeO_2_ was anchored on the 3D hierarchical nitrogen‐doped porous carbon (3D‐CeO_2_@CN) and used for FA oxidation.^[^ ^10c^ ^]^ In this case, CeO_2_ provided a high number of oxygen vacancies, abundant active surface oxygen, and high reducibility. Electron transfer from the N atoms of the surface CN further resulted in more oxygen defects and surface oxygen on CeO_2_ compared to pure CeO_2_. The 3D hierarchical structure of CN helped stabilize CeO_2_, facilitating the mass transfer of FA molecules. As such, the prepared 3D‐CeO_2_@CN was able to completely oxidize 90 ppm FA at 170 °C, which was about 130 °C lower than that of pure CeO_2_ catalysts at a GSHV of 100 L g^−1^ h^−1^.^[^ ^10c^ ^]^ The surface functionalization of the matrix is regarded as an effective approach to enhance the compatibility of TMOs with nanomaterial matrices.^[^ ^41b^ ^]^ For example, the carboxyl and hydroxyl functionalization of a polyethylene terephthalate (PET) surface (i.e., matrix) helped form a firmly attached thin MnO*_x_* layer on the surface of PET fibers. The synthesized MnO*_x_*/PET composite showed no agglomeration of powder MnO*_x_* catalysts with the reduced air pressure drop. In addition, the formulated composite was capable of degrading ≈94% of FA (0.6 mg m^−3^) at room temperature.^[^ ^41b^ ^]^

**Figure 6:**
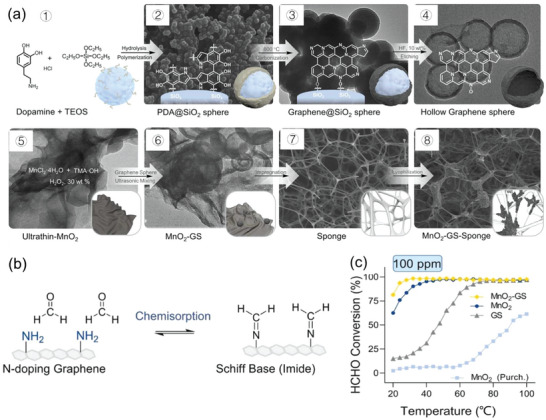
The oxidation of FA by MnO_2_‐graphene sphere (GS)‐Sponge: a) The synthesis process of MnO_2_‐GS‐sponge, b) the chemisorption of FA onto MnO_2_‐GS‐sponge through the formation of imide products, and c) FA (100 ppm) conversion over MnO_2_‐GS‐sponge catalysts and its counterparts as a function of temperature, Reproduced with permission.^[^ ^10a^ ^]^ Copyright 2023, Elsevier.

Graphene has also been employed to couple with TMOs for accelerating charge transport during redox processes against FA using its exceptional electron conductivity.^[^ ^26a^ ^]^ In comparison to the pure MnO_2_, the 2D structure of graphene can facilitate the adsorption of FA and O_2_ molecules to expose more active sites for catalysis. Hybridized areas of graphene‐MnO_2_ provide important interfaces where the conducting graphene greatly accelerated the charge transfer between Mn^4+^ and Mn^3+^ species.^[^ ^26a^ ^]^ As such, the catalytic performance of MnO_2_/graphene hybrids achieved completed conversion of FA (100 ppm) at 65 °C, which lasted up to 70 h.^[^ ^26a^ ^]^ Likewise, the heterostructure of nanosheet MnO_2_ encapsulating N‐doped graphene spheres (GS) was deposited in a network‐like sponge (acting as support) to prepare 3D structure MnO_2_‐GS sponge composites for FA oxidation (**Figure** **6**a).^[^ ^10a^ ^]^ Such a 3D structure facilitated the catalytic degradation of FA by exposing more active sites to FA molecules. The enhanced potential of MnO_2_‐GS sponge composites was also reported, as it could favorably adsorb FA molecules by amino groups on N‐doped GS surface through the formation of imide products (Figure 6b).^[^ ^10a^ ^]^ Accordingly, the MnO_2_‐GS sponge composites showcased 96.7% conversion of FA at low temperature (<35 °C), which was far better than that of the pristine MnO_2_ nanosheets (95.3% conversion at 40 °C) (Figure 6c).^[^ ^10a^ ^]^ In addition, N‐doped carbon nanotubes (NCNT) have also received interest because of their large surface reactive sites suitable for high catalytic efficiency.^[^ ^47^ ^]^ For instance, NCNT increased the number of structural defects with increases in electron transfer at the interfaces between NCNT and MnO_2_ of MnO_2_/NCNT composites.^[^ ^48^ ^]^ In addition, oxygen molecules were readily activated on NCNT through the formation of active superoxide species to promote the regeneration of MnO_2_. As such, the prepared MnO_2_/NCNT composites showed good activity and selectivity for FA oxidation.^[^ ^48b^ ^]^

## 4 Factors Influencing the Catalytic Activity of TMO‐Based Thermocatalysts

The catalytic efficiency of TMO‐based thermocatalysts can be influenced by several factors such as crystal form, catalyst preparation process (e.g., synthesis methods and calcination temperature), physiochemical properties (e.g., morphology, surface area, and particle sizes), and other experimental operation parameters (e.g., formaldehyde feed rate and humidity levels).^[^ ^3^, ^5^ ^]^ Several articles have reviewed the effect of such factors on catalytic performances of TM‐based thermocatalysts.^[^ ^3^, ^4^, ^5^, ^49^ ^]^ For instance, the effects of preparation method, morphology, specific area, and experimental parameters (e.g., water vapor content, initial FA concentration, and space velocity) were described with respect to the thermocatalytic performance of TMO catalysts.^[^ ^49b^ ^]^ In addition, the effect of crystal form, surface morphology, microstructure, and temperature on the FA thermocatalytic performance of manganese oxides catalysts was also discussed.^[^ ^5a^ ^]^ The catalytic performance of TMOs against FA was also significantly affected by many other factors such as exposed crystal facets, metallic/non‐metallic modifications, type of precursors, alkali/acid treatment, and type of matrix/substrate. Hence, the following sections are organized to address the various factors that can influence thermocatalytic performance of TM‐based materials.

### 4.1 Effect of Exposed Crystal Facets on Catalytic Activity

**Figure 7:**
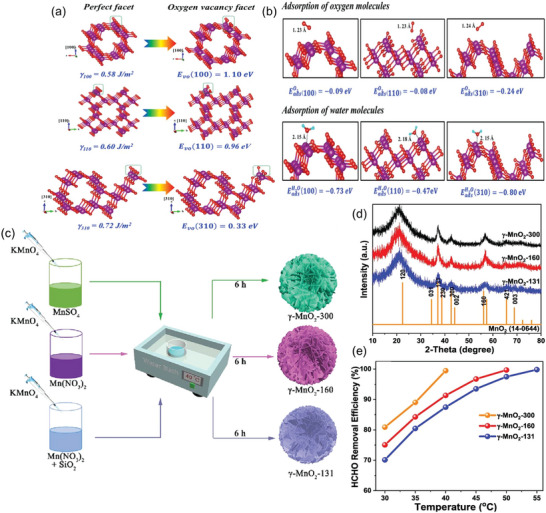
The exposed facets of different MnO_2_ structures and their effects on FA oxidation: *α*‐MnO_2_: a) Surface energy and formation energy of oxygen vacancy and b) adsorption energy of oxygen and water molecules, Reproduced with permission.^[^ ^28a^ ^]^ Copyright 2018, American Chemical Society (ACS). *γ*‐MnO_2_: c) Illustration of synthesis procedure, d) XRD patterns, and e) FA removal efficiency between different exposed facets, Reproduced with permission.^[^ ^52^ ^]^ Copyright 2023, Elsevier.

The exposed specific crystal facets of TMO‐based thermocatalysts are an important variable to control the FA oxidation potential through adsorption/activation of O_2_ and H_2_O, as they can accommodate abundant TM cations for the generation of OVs.^[^ ^13^, ^28^, ^50^ ^]^ For this purpose, various single crystalline *α*‐MnO_2_ with exposed facets of (3 1 0), (1 1 0), and (1 0 0) were prepared by facile hydrothermal redox reaction using KMnO_4_/(NH_4_)_2_C_2_O_4_·H_2_O, KMnO_4_/(NH_4_)_2_SO_4_, and MnSO_4_·H_2_O/(NH_4_)_2_S_2_O_8_/(NH_4_)_2_SO_4_ as reactants, respectively.^[^ ^28a^ ^]^ As a result, the prepared *α*‐MnO_2_ nanowires with high index exposed (3 1 0) exhibited better catalytic performance for the complete degradation of 100 ppm FA under a gas hourly space velocity (GHSV) of 90 L g^−1^ h^−1^ at 60 °C when compared to (1 1 0) (at 130 °C) and (1 0 0) (at 150 °C).^[^ ^28a^ ^]^ The superiority of exposed (3 1 0) facets of *α*‐MnO_2_ is supported by its highest surface energy (0.72 J m^−2^) and lowest energy for the formation of oxygen vacancies (0.33 eV) (**Figure** **7**a). Enhanced absorptivity against atmospheric oxygen (in terms of adsorption energy) onto the exposed (3 1 0) facets (−0.24 eV) (e.g., relative to that of (1 1 0) (−0.08 eV) and (1 0 0) facets (−0.09 eV)) is also beneficial to rapidly replenish lattice oxygen for catalytic oxidation of FA (Figure 7b). Meanwhile, such exposed (3 1 0) facets also showed enhanced water molecule adsorption for recharging the consumed surface hydroxyls to facilitate FA adsorption onto *α*‐MnO_2_ via hydrogen bond formation.^[^ ^28^, ^33^, ^51^ ^]^ As such, the single crystalline *α*‐MnO_2_ nanowires with exposed (3 1 0) facets showed the highest activity for FA oxidation.^[^ ^28a^ ^]^ Similarly, three *γ*‐MnO_2_ catalysts with the exposed facets of (3 0 0), (1 6 0), and (1 3 1) were synthesized and tested for FA removal (Figure 7c,d).^[^ ^52^ ^]^ *γ*‐MnO_2_ with exposed (3 0 0) facet (i.e., *γ*‐MnO_2_‐300) had the best FA oxidation activity such as 100% elimination of 100 ppm FA under GHSV of 300 L g^−1^ h^−1^ at around 40 °C when compared to (1 6 0) (at 50 °C) and (1 3 1) facets (at 55 °C) (Figure 7e). The excellent FA catalytic activity of *γ*‐MnO_2_‐300 was attributable to the high surface area, abundant active surface lattice oxygen, and oxygen vacancy.^[^ ^52^ ^]^

In another study, the (1 1 0) facet of Co_3_O_4_ with sufficient Co^3+^ was also found to be the most effective plane for FA oxidation relative to other facets (e.g., (2 2 0)).^[^ ^13a^ ^]^ This is because Co^3+^ of Co_3_O_4_ (1 1 0) facets exhibited strong ability to dissociate FA (Co^3+^ + HCHO→ Co^2+^ + ●CHO + H^+^) with good affinity toward FA and O_2_ molecules.^[^ ^13^, ^50^ ^]^ Moreover, the adsorbed O_2_ could also be readily converted into active oxygen species (e.g., O_2_^2−^) on the Co_3_O_4_ (1 1 0) facets to increase FA catalytic activity.^[^ ^50a^ ^]^

### 4.2 Effect of Alkali Metal/Nitrogen Modification on Catalytic Activity

The introduction of alkali metal/nitrogen has been reported for enhancing the catalytic activity of oxides materials by imparting abundant OVs and enhancing FA adsorption.^[^ ^53^ ^]^ As such, the modification of TMO‐based thermocatalysts with metal‐like alkali metal (e.g., K^+^) and nitrogen is an effective option to enhance the formation of abundant OVs, to facilitate adsorbed O_2_ activation, and to promote the adsorption of FA molecules for the effective thermocatalysis of FA.^[^ ^53^, ^54^ ^]^ For instance, the introduction of K^+^ ions over Co_3_O_4_ catalysts both increased the proportion of Co^3+^/Co^2+^ (0.42) relative to pure Co_3_O_4_ (0.30) and improved the number of OVs on the surface of modified Co_3_O_4_. This further boosted the oxidation ability of the modified thermocatalysts toward FA molecules.^[^ ^54d^ ^]^ As a result, the K^+^ modified Co_3_O_4_ was able to completely convert 100 ppm FA to CO_2_ at a lower temperature (i.e., 100 °C) than the pure Co_3_O_4_ (120 °C).^[^ ^54d^ ^]^ Interestingly, the isolated and localized K^+^ modification of layered MnO_2_ exhibited a distinct effect on FA removal for the generated thermocatalyst.^[^ ^54a^ ^]^ As the isolated K^+^ was dissociated between layers via weak chemical bonds, the desorption of H_2_O molecules was restricted to hinder the FA catalytic process. In contrast, the coordination of localized K^+^ with oxygen atoms at the vacancy sites was a useful option to activate O_2_ with poor adsorption of H_2_O molecules. As such, the FA catalytic activity of the resulting thermocatalyst was enhanced in a localized K^+^ modified layer MnO_2_.^[^ ^54a^ ^]^ Similar alkali‐promoted effects were also observed for noble metal supported catalysts (e.g., K‐Ag/Al_2_O_3_).^[^ ^55^ ^]^ Recently, the nitrogen modified *α*‐MnO_2_ was reported to enhance the thermocatalytic decomposition of FA by achieving complete oxidation of 60 ppm FA under a GHSV of 90 L g^−1^ h^−1^ at 90 °C.^[^ ^54e^ ^]^ In this respect, the interstitial sites of thermocatalysts (Mn‐N‐O or Mn‐O‐N) were found to be the most suitable sites for introducing non‐metal nitrogen on *α*‐MnO_2_. This was due to the potential of such interstitial sites for OV formation as well as FA/O_2_ adsorption/activation, which are beneficial for FA oxidation.^[^ ^54e^ ^]^

### 4.3 Effect of Precursors on Catalytic Activity

**Figure 8:**
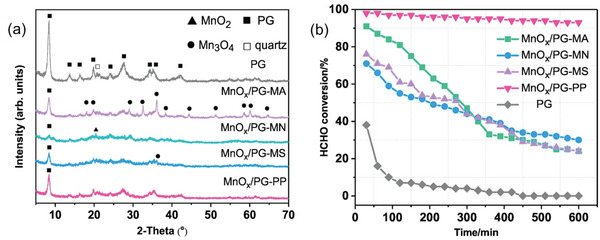
The effect of four precursors (e.g., manganese acetate (MA), manganese nitrate (MN), manganese sulfate (MS), and potassium permanganese (PP)) on FA catalytic activity of palygorskite (PG)‐supported manganese oxide (MnO*_x_*/PG) catalysts: a) XRD patterns and b) FA conversions over palygorskite (PG)‐supported manganese oxide (MnO*_x_*/PG) catalysts prepared by diverse precursors, Reproduced with permission.^[^ ^56c^ ^]^ Copyright 2019, Elsevier.

The utilization of different precursors in the preparation of TMO‐based catalysts was also found to influence the redox ability, the amount of active oxygen species, crystal forms, and physiochemical properties.^[^ ^56^ ^]^ The utilization of bases during the synthesis of TMO‐based catalysts promoted their FA catalytic oxidation properties through increases in their OH^−^ content and surface area.^[^ ^57^ ^]^ As such, the effect of alkali precipitants (e.g., NH_3_·H_2_O, KOH, NH_4_HCO_3_, K_2_CO_3_, and KHCO_3_) was investigated with regard to the thermal catalytic activity of Co_3_O_4_ catalysts toward gaseous FA.^[^ ^56a^ ^]^ The Co_3_O_4_ synthesized using KHCO_3_ possessed abundant hydroxyl groups with a high ratio of Co^3+^/Co^2+^, exhibiting high catalytic activity against gaseous FA. Moreover, the BET specific areas and pore volumes of KHCO_3_ derived Co_3_O_4_ (97.9 m^2^ g^−1^/0.411 cm^3^ g^−1^) were higher than that of Co_3_O_4_ prepared by NH_3_·H_2_O (67.7 m^2^ g^−1^/0.135 cm^3^ g^−1^), KOH (54.8 m^2^ g^−1^/0.105 cm^3^ g^−1^), NH_4_HCO_3_ (95.4 m^2^ g^−1^/0.393 cm^3^ g^−1^), and K_2_CO_3_ (78.7 m^2^ g^−1^/0.337 cm^3^ g^−1^). Such larger specific areas and pore volumes of KHCO_3_ derived Co_3_O_4_ can also facile the FA transportation onto active sites for oxidation, thereby enhancing the FA oxidation process.^[^ ^56a^ ^]^ The role of manganese ions as precursors on the crystal forms of palygorskite (PG)‐supported manganese oxides (MnO*_x_*/PG) was investigated in another study.^[^ ^56c^ ^]^ Accordingly, the utilization of manganese acetate (MA) resulted in the formation of amorphous Mn_2_O_3_ and a crystalline Mn_3_O_4_ mixture. The utilization of manganese nitrate (MN) yielded *β*‐MnO_2_, while manganese sulfate (MS) formed a mixture of Mn_3_O_4_ and low crystalline *γ*‐MnO_2_. In contrast, low crystalline *δ*‐MnO_2_ was obtained when potassium permanganese (PP) was used as a manganese ionic source (**Figure** **8**a). Among these, the MnO*_x_*/PG‐PP possessed large specific surface area, crystalline *δ*‐MnO_2_, highly distributed active components (e.g., surface active oxygen species), high content of Mn^4+^ species, and increased lattice oxygen content.^[^ ^56c^ ^]^ Consequently, the MnO*_x_*/PG‐PP exhibited the best catalytic activity (95% removal of 1 ppm FA at 25 °C) even after 600 min among all other MnO*_x_*/PG‐(MA, MS, and MN) catalysts (below 30% after 600 min) (Figure 8b).^[^ ^56c^ ^]^

### 4.4 Effect of Alkali/Acid Treatment on Catalytic Activity

**Figure 9:**
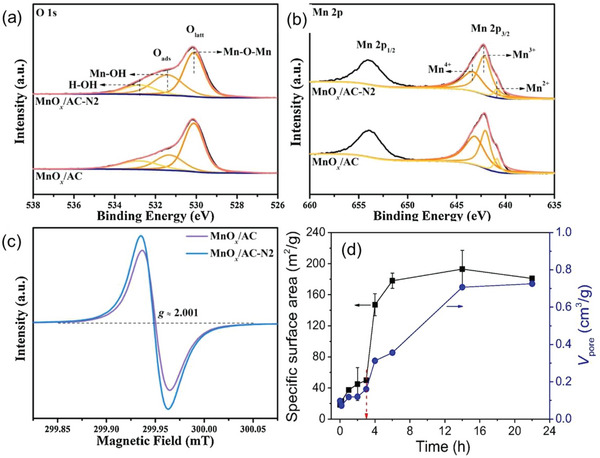
The alkali treatment effect on catalytic activity of *δ*‐MnO*_x_* supported by AC (*δ*‐MnO*_x_*/AC) catalysts: a) O 1s XPS spectra, b) Mn 2p XPS spectra, and c) (EPR) spectra of *δ*‐MnO*_x_*/AC and *δ*‐MnO*_x_*/AC‐N2 (prepared by 0.07 mol ammonia), Reproduced with permission.^[^ ^23b^ ^]^ Copyright 2020, Elsevier. d) Specific surface area and total pore volume of *ε*‐MnO_2_ catalyst changes upon time of acid treatment. Reproduced with permission.^[^ ^59b^ ^]^ Copyright 2020, Elsevier.

Alkali/acid treatments can be used to improve the FA catalytic performance of TMO‐based catalysts through the introduction of —OH groups, defect sites, and OVs.^[^ ^13^, ^23^, ^58^ ^]^ For instance, the post alkali (NaOH) treatment of NiCo_2_O_4_ endowed it with more hydroxyl groups and Co^3+^/Ni^2+^ content compared to the untreated NiCo_2_O_4_.^[^ ^13b^ ^]^ In another study, birnessite‐type MnO_2_ treated by tetrabutylammonium hydroxide exhibited a lower apparent activation energy for FA oxidation than the pristine MnO_2_.^[^ ^58^ ^]^ This is because tetrabutylammonium hydroxide helped generate irregular surface pits on MnO_2_, which both provided larger specific surface areas and led to the formation of more high valent manganese species (e.g., Mn^4+^ and Mn^3+^) and surface oxygen. As a result, the FA adsorption and oxidation properties of MnO_2_ were enhanced considerably.^[^ ^58^ ^]^ Likewise, ammonia was also utilized to control the OV formation onto *δ*‐MnO*_x_* supported by AC (*δ*‐MnO*_x_*/AC) for the enhancement of FA catalytic removal.^[^ ^23b^ ^]^ Accordingly, the *δ*‐MnO*_x_*/AC‐N_2_ (ammonia used at 0.07 mol) had recorded higher ratio values for adsorbed oxygen (O_ads_)/lattice oxygen (O_latt_) (0.71) and Mn^3+^/Mn^4+^ (1.25) than those of pristine *δ*‐MnO*_x_*/AC (0.44 and 0.73, respectively) (**Figure** **9**a,b), which supports the suitable conditions for the favorable formation of OVs. As such, an increased relative abundance of OVs in *δ*‐MnO*_x_*/AC‐N_2_ (i.e., compared with *δ*‐MnO*_x_*/AC) was evident in electron paramagnetic resonance (EPR) spectra (Figure 9c). The abundant OVs were thus helpful to improve the catalytic activity of *δ*‐MnO*_x_*/AC‐N_2_ against FA as they promoted the adsorption, activation, and migration of oxygen molecules to form more active oxygen species (e.g., O_2_^●^).^[^ ^23b^ ^]^

The surface structure and redox properties of TMO‐based catalysts can also be affected by acid treatment.^[^ ^8^, ^59^ ^]^ In particular, the H_2_SO_4_ treatment of MnO*_x_*‐CeO_2_ catalysts showed higher surface area (310%) and stronger redox properties than their untreated counterparts.^[^ ^8^ ^]^ It was observed that the textural and redox properties of MnO*_x_*‐CeO_2_ samples were altered considerably when the Mn content was above 50%. The higher surface area of acid treated thermocatalysts may reflect the dissolution of Mn^2+^ ions by H_2_SO_4_ acid. In addition, acid treatment oxidized the manganese species to a higher oxidation state via Mn dismutation reaction (Mn_3_O_4_ + 4H^+^ = MnO_2_ + 2Mn^2+^ + 2H_2_O) improving the redox properties of catalysts.^[^ ^8^ ^]^ The extension of acid treatment time can also improve textual properties (e.g., BET surface area and pore volume) of the *ε*‐MnO_2_ catalyst while developing interconnected macro‐mesoporous networks^[^ ^59b^ ^]^ (Figure 9d). The BET surface area/pore volume of the resulting materials increased from 18 m^2^ g^−1^/0.1 cm^3^ g^−1^ to 181 m^2^ g^−1^/0.73 cm^3^ g^−1^ as the acid treatment duration increased from 0 h to 22 h. Such alteration of *ε*‐MnO_2_ catalyst led to the noticeable improvement in the FA catalytic activity.^[^ ^59b^ ^]^

## 5 Performance Comparison of TM‐Based Catalyst for FA Degradation

Quantitative evaluation of thermocatalytic performance of TMO‐based catalysts is important to properly assess their practical feasibility toward FA oxidation. In this regard, the catalytic performances of different types of TMO‐based catalysts were compared in terms of the key metrics (e.g., T_90_ and kinetic reaction rate) as summarized in Tables 1 and **2**. Note that T_90_ value is the temperature corresponding to 90% removal efficiency of FA gas over TMO‐based catalysts.^[^ ^26b^ ^]^ The kinetic reaction rate (r, equation 1) is also a useful tool to offer a meaningful comparison between different catalytic systems as it can be used to integrate the interaction between important process variables (e.g., catalyst mass, pollutant conversion level, pollutant concentration, and pollutant feeding rate).^[^ ^60^ ^]^

(1) $$\begin{array}{ccc} & & \mathrm{Reaction}\;\mathrm{rate}\left(r,{\;\mathrm{mmol}\;\mathrm{mg}}^{-1}h^{-1}\right)\\ & & =\frac{10^{3}\;\times \;3600\;\times \;\mathrm{conversion}\;\left(\%\right)\;\times \;\mathrm{flow}\;\mathrm{rate}\;\left({\mathrm{mol}\;s}^{-1}\right)}{\mathrm{catalyst}\;\mathrm{mass}\;\left(\mathrm{mg}\right)}\; \end{array}$$

To make this comparison more meaningful, all TMO‐based catalysts (mono‐metallic, bi‐metallic, and TMO‐based composites) have been classified into two categories based on their working temperature as low (room temperature) and high temperature (>100 °C) thermocatalysts (Tables 1 and 2). As such, the kinetic reaction rate (*r*) values at room temperature (RT) and at high temperature (e.g., 100 °C) are utilized to compare their catalytic performances.

**Table 2:** Summary of transition metal based thermocatalysts for catalytic degradation of FA under high temperature conditions

Order	Catalyst	Preparation method	Catalyst mass [mg]	Reactant mixture	Pollutant concentration [ppm]	Flow rate [mL min^−1^]	Flow rate [mol s^−1^]	Space velocity	T_90_ [°C]	Conversion [%] at 100 °C	r [mmol mg_cat_^−1^ h^−1^]^c^	BET [m^2^ g^−1^]	Reference
[A] Mono transition metal oxide‐based thermocatalysts													
1	Alkali treated Birnessite	Redox reaction	100	200 ppm FA + air 45%RH	200	200	2.73E‐08	120 000 mL g^−1^ h^−1^	86	100	9.82E‐02	128.8	[58]
2	Birnessite	Redox reaction	100	40 ppm FA + 80% RH + Air	40	200	5.45E‐09	120 000 mL g^−1^ h^−1^	96	98	1.92E‐02	14.4	[69]
3	Birnessite	Hydrothermal method	100	460 ppm FA + purified Air	460	50	1.57E‐08	30 000 mL g^−1^ h^−1^	97	100	5.64E‐02	154	[70]
5	Birnessite	Hydrothermal method	50	100 ppm FA + 20% O_2_ + He	100	50	3.41E‐09	50 000 mL g^−1^ h^−1^	120	61	1.50E‐02	NA	[71]
6	Cryptomelane	Hydrothermal method	50	100 ppm FA + 20% O_2_ + He	100	50	3.41E‐09	50 000 mL g^−1^ h^−1^	120	76	1.87E‐02	94.6	[71]
7	Cryptomelane	Hydrothermal method	200	400 ppm FA + 10% O_2_ + N_2_	400	100	2.73E‐08	18 000 mL g^−1^ h^−1^	128	20	9.82E‐03	63.4	[72]
8	Pyrolusite	Hydrothermal method	200	400 ppm FA + 10% O_2_ + N_2_	400	100	2.73E‐08	18 000 mL g^−1^ h^−1^	174	5	2.45E‐03	32.0	[72]
9	Ramsdellite	Hydrothermal method	50	100 ppm FA + 20% O_2_ + He	100	50	3.41E‐09	50 000 mL g^−1^ h^−1^	150	52	1.28E‐02	NA	[71]
10	Todorokite	Hydrothermal method	200	400 ppm FA + 10% O_2_ + N_2_	400	100	2.73E‐08	18 000 mL g^−1^ h^−1^	155	10	4.91E‐03	79.7	[72]
11	OMS‐2	Refluxing method	200	500 ppm FA + 10% O_2_ + N_2_	500	100	3.41E‐08	30 000 mL g^−1^ h^−1^	110	40	2.45E‐02	68	[73]
12	K‐OMS‐2	Sol‐gel reaction	100	460 ppm FA + 21% O_2_ + N_2_	460	50	1.57E‐08	30 000 mL g^−1^ h^−1^	150	28	1.58E‐02	206	[74]
13	K‐OMS‐2	Soft chemistry	100	460 ppm FA + purified air	460	50	1.57E‐08	20 000 mL g^−1^ h^−1^	NA	64	3.61E‐02	33.7	[75]
14	Mesoporous MnO*_x_*	Wet‐chemistry	200	500 ppm FA + 20% O_2_ + He	500	NA	NA	NA	NA	NA	NA	121	[59a]
15	Nest‐like MnO_2_	Hydrothermal method	100	460 ppm FA + purified air	460	50	1.57E‐08	20 000 mL g^−1^ h^−1^	152	40	2.26E‐02	56.9	[35]
16	Urchin‐like MnO_2_	Hydrothermal method	100	460 ppm FA + purified air	460	50	1.57E‐08	20 000 mL g^−1^ h^−1^	160	32	1.81E‐02	62.3	[35]
17	Cocoon‐like MnO_2_	Hydrothermal method	100	460 ppm FA + purified air	460	50	1.57E‐08	20 000 mL g^−1^ h^−1^	185	28	1.58E‐02	247.6	[35]
18	*α*‐MnO_2_ nanorods	Hydrothermal method	200	400 ppm FA + 20% O_2_ + N_2_	400	100	2.73E‐08	30 000 mL g^−1^ h^−1^	130	75	3.68E‐02	NA	[33c]
19	*α*‐MnO_2_ Nanowire	Hydrothermal method	100	100 ppm FA + 21%O_2_ + N_2_	100	150	1.02E‐08	90 000 mL g^−1^ h^−1^	55	100	3.68E‐02	135.3	[28a]
20	*α*‐MnO_2_	Hydrothermal method	60	170 ppm FA + 20% O_2_ + 25% RH + N_2_	170	100	1.16E‐08	100 000 mL g^−1^ h^−1^	112	70	4.87E‐02	80.8	[19]
21	*β*‐MnO_2_	Hydrothermal method	200	400 ppm FA + 20% O_2_ + N_2_	400	100	2.73E‐08	30 000 mL g^−1^ h^−1^	165	1	4.91E‐04	NA	[33c]
22	*β*‐MnO_2_	Hydrothermal method	60	170 ppm FA + 20% O_2_ + 25% RH + N_2_	170	100	1.16E‐08	100 000 mL g^−1^ h^−1^	173	8	5.56E‐03	23.3	[19]
23	*γ*‐MnO_2_	Hydrothermal method	60	170 ppm FA + 20% O_2_ + 25% RH + N_2_	170	100	1.16E‐08	100 000 mL g^−1^ h^−1^	145	20	1.39E‐02	85.3	[19]
24	*δ*‐MnO_2_	Hydrothermal method	60	170 ppm FA + 20% O_2_ + 25% RH + N_2_	170	100	1.16E‐08	100 000 mL g^−1^ h^−1^	74	100	6.95E‐02	108.4	[19]
25	*ε*‐MnO_2_	Acid etching	200	100 ppm FA + 20% O_2_ + He	100	100	6.82E‐09	30 000 mL g^−1^ h^−1^	141	50	6.13E‐03	181	[59b]
26	K modified MnO_2_	Hydrothermal method	100	200 ppm FA + air	200	200	2.73E‐08	120 000 mL g^−1^ h^−1^	98	94	9.23E‐02	88	[54a]
27	3D‐MnO_2_	Freezing method	50	100 ppm FA + air + 65%RH	100	150	1.02E‐08	180 000 mL g^−1^ h^−1^	84	100	7.36E‐02	99.8	[33b]
28	3D mesoporous‐ MnO_2_	Template method	200	400 ppm FA + 20% O_2_ + N_2_	400	100	2.73E‐08	30 000 mL g^−1^ h^−1^	110	76	3.73E‐02	NA	[33c]
29	MnO_2_ nanosheets	One‐step method	50	100 ppm FA + air + 65%RH	100	150	1.02E‐08	180 000 mL g^−1^ h^−1^	144	36	2.65E‐02	202.9	[33b]
30	MnO_2_ nanowires	Hydrothermal method	50	100 ppm FA + air + 65%RH	100	150	1.02E‐08	180 000 mL g^−1^ h^−1^	154	22	1.62E‐02	16.7	[33b]
31	2D‐Co_3_O_4_	Template method	200	400 ppm FA + 20%O_2_ + N_2_	400	100	2.73E‐08	30 000 mL g^−1^ h^−1^	140	8	3.93E‐03	43.3	[29b]
32	3D‐Co_3_O_4_	Precipitation method	200	400 ppm FA + 20%O_2_ + N_2_	400	100	2.73E‐08	30 000 mL g^−1^ h^−1^	120	30	1.47E‐02	85.9	[29b]
33	3D‐Co_3_O_4_	Template method	200	100 ppm FA + 20%O_2_ + N_2_	100	100	6.82E‐09	30 000 h^−1^	100	90	1.10E‐02	87.8	[57b]
34	Co_3_O_4_ nanofibers	Electrospinning and calcination	100	400 ppm FA + air	400	50	1.36E‐08	30 000 mL g^−1^ h^−1^	95	100	4.91E‐02	561.32	[76]
35	Co_3_O_4_ cube	Hydrothermal method	100	100 ppm FA + 20%O_2_ + N_2_	100	100	6.82E‐09	69 000 h^−1^	136	5	1.23E‐03	3.7	[28b]
36	Co_3_O_4_ rod	Precipitation method	100	100 ppm FA + 20%O_2_ + N_2_	100	100	6.82E‐09	69 000 h^−1^	100	90	2.21E‐02	83.5	[28b]
37	Co_3_O_4_ sheet	Hydrothermal method	100	100 ppm FA + 20%O_2_ + N_2_	100	100	6.82E‐09	69 000 h^−1^	118	25	6.13E‐03	8.5	[28b]
38	K‐ Co_3_O_4_	Precipitation method	100	100 ppm FA + 21%O_2_ + N_2_	100	100	6.82E‐09	69 000 h^−1^	89	100	2.45E‐02	70.9	[54d]
39	Nano Co_3_O_4_	Precipitation method	200	400 ppm FA + 20% O_2_ + N_2_	400	100	2.73E‐08	30 000 mL g^−1^ h^−1^	225	1	4.91E‐04	28.1	[29b]
40	Spinel Co_3_O_4_	Precipitation method	100	100 ppm FA + 21% O_2_ + N_2_	100	100	6.82E‐09	69 000 h^−1^	87	100	2.45E‐02	97.9	[56a]
41	CeO_2_	Electrodeposition method	200	50 ppm FA + 25% O_2_ + N_2_	50	100	3.41E‐09	30 000 mL g^−1^ h^−1^	250	8	4.91E‐04	12.53	[16a]
42	CeO_2_ nanospheres	Hydrothermal method	50	810 ppm FA + 20% O_2_ + N_2_	810	100	5.52E‐08	84 000 h^−1^	220	8	3.18E‐02	57.8	[77]
43	CeO_2_ spherical like	Hydrothermal method	240	500 ppm FA + 20% O_2_ + N_2_	500	40	1.36E‐08	10 000 mL g^−1^ h^−1^	120	85	1.74E‐02	86	[34]
44	CeO_2_ nanorod	Hydrothermal method	240	500 ppm FA + 20% O_2_ + N_2_	500	40	1.36E‐08	10 000 mL g^−1^ h^−1^	180	65	1.33E‐02	44	[34]
45	CeO_2_ nanocubes	Hydrothermal method	240	500 ppm FA + 20% O_2_ + N_2_	500	40	1.36E‐08	10 000 mL g^−1^ h^−1^	NA	30	6.13E‐03	11	[34]
46	CeO_2_ bulk particles	Thermal decomposition	50	810 ppm FA + 20% O_2_ + N_2_	810	100	5.52E‐08	84 000 h^−1^	NA	5	1.99E‐02	148.6	[77]
47	NiO	Nanocasting method	100	60 ppm FA + 21% O_2_ + N_2_ + O_3_ + air	60	100	4.09E‐09	60 000 mL g^−1^ h^−1^	80	100	1.47E‐02	98	[78]
48	3D‐Cr_2_O_3_	Impregnated method	100	500 ppm FA + 20% O_2_ + N_2_	500	50	1.70E‐08	30 000 mL g^−1^ h^−1^	118	65	3.99E‐02	124	[27b]
[B] Bi‐transition metal oxide‐based thermocatalysts													
49	3D‐Co‐Mn	Template method	250	80 ppm FA + 21% O_2_ + N_2_ + 50%RH	80	100	5.45E‐09	36 000 h^−1^	68	100	7.85E‐03	150	[36b]
50	3D‐Co‐Mn	Co‐precipitation method	250	80 ppm FA + 21% O_2_ + N_2_ + 50%RH	80	100	5.45E‐09	36 000 h^−1^	74	100	7.85E‐03	92	[36b]
51	Mn*_x_* Co_3−_ *_x_* O	Co‐precipitation method	150	80 ppm FA + 21% O_2_ + N_2_ + 50%RH	80	100	5.45E‐09	60 000 h^−1^	74	100	1.31E‐02	157	[39a]
52	Mn*_x_* Co_3−_ *_x_* O	Citric acid method	150	80 ppm FA + 21% O_2_ + N_2_ + 50%RH	80	100	5.45E‐09	60 000 h^−1^	98	100	1.31E‐02	92	[39a]
53	Ce‐MnO_2_	Redox reaction	100	190 ppm FA + 20% O_2_ + N_2_	190	150	1.94E‐08	90 000 mL g^−1^ h^−1^	90	100	6.99E‐02	129	[18]
54	Mn_0.5_Ce_0.5_O_2_	Pechini method	300	33 ppm FA + 21%O_2_ + air	33	600	1.35E‐08	10 000 h^−1^	250	10	1.62E‐03	90.1	[37b]
55	MnO*_x_*‐CeO_2_	Sol‐gel method	200	580 ppm FA + 18%O_2_ + He	580	100	3.95E‐08	21 000 mL g^−1^ h^−1^	177	NA	NA	201.2	[36a]
56	MnO*_x_*‐CeO_2_	Co‐precipitation method	200	580 ppm FA + 18%O_2_ + He	580	100	3.95E‐08	21 000 mL g^−1^ h^−1^	150	NA	NA	126.3	[36a]
57	MnO*_x_*‐CeO_2_	Modified co‐precipitation method	200	580 ppm FA + 18%O_2_ + He	580	100	3.95E‐08	21 000 mL g^−1^ h^−1^	90	100	7.12E‐02	124	[36a]
58	MnO*_x_*‐CeO_2_	Co‐precipitation method	200	580 ppm FA + 20% O_2_ + N_2_	580	100	3.95E‐08	30 000 mL g^−1^ h^−1^	110	84	5.98E‐02	66	[79]
59	MnO*_x_*‐CeO_2_	Co‐precipitation method	200	400 ppm FA + 20%O_2_ + He	400	100	2.73E‐08	30 000 mL g^−1^ h^−1^	140	48	2.36E‐02	160	[8]
60	MnO*_x_*‐SnO_2_	Redox co‐precipitation method	200	400 ppm FA + 10% O_2_ + N_2_	400	100	2.73E‐08	30 000 mL g^−1^ h^−1^	174	NA	NA	136.9	[80]
61	MnO*_x_*‐SnO_2_	Co‐precipitation method	200	400 ppm FA + 10% O_2_ + N_2_	400	100	2.73E‐08	30 000 mL g^−1^ h^−1^	215	NA	NA	132.2	[80]
62	CeO_2_‐Co_3_O_4_	Sol‐gel method	50	100 ppm FA + 21% O_2_ + N_2_	100	60	4.09E‐09	36 000 mL g^−1^ h^−1^	60	100	2.94E‐02	61.8	[37a]
63	Ni_0.8_Co_2.2_O_4_	Co‐precipitation method	100	100 ppm FA + 21% O_2_ + N_2_	100	100	6.82E‐09	69 000 h^−1^	86	100	2.45E‐02	NA	[40]
64	Co_3_O_4_/CoMn_2_O_4_	Template method	100	80 ppm FA + 20% O_2_ + N_2_ + 60%RH	80	100	5.45E‐09	60 000 mL g^−1^ h^−1^	106	72	1.41E‐02	124.5	[22]
65	Co*_x_* Mn_3‐_ *_x_* O_4_	Electrodeposition	850	50 ppm FA + 25% O_2_ + N_2_ + 50%RH	50	200	6.82E‐09	120 000 mL g^−1^ h^−1^	90	100	2.89E‐03	11.6	[38]
66	Co_3_O_4_/ZrO_2_	Co‐precipitation method	200	150 ppm FA + 20%O_2_ + He	150	100	1.02E‐08	30 000 mL g^−1^ h^−1^	170	NA	NA	60.6	[56b]
67	Eu‐CeO_2_	Electrodeposition method	200	50 ppm FA + 25% O_2_ + N_2_	50	100	3.41E‐09	30 000 mL g^−1^ h^−1^	125	62	3.80E‐03	28.24	[16a]
[C] Transition metal oxide‐based composites thermocatalysts													
68	Co_3_ *_x_* Mn*_x_* O_4_‐carbon	Impregnated method	30	90 ppm FA + air	90	20	1.23E‐09	40 000 mL g^−1^ h^−1^	85	100	1.47E‐02	NA	[44c]
69	MnO_2_/N‐doped carbon nanotube	Impregnated method	200	100 ppm FA	100	100	6.82E‐09	30 000 mL g^−1^ h^−1^	NA	100	1.23E‐02	85.52	[48b]
70	Graphene/MnO_2_	Hummer's method	100	100 ppm FA + air	100	50	3.41E‐09	30 000 mL g^−1^ h^−1^	62	100	1.23E‐02	71	[26a]
71	Graphene/3D‐MnO_2_	In situ synthesis method	250	100 ppm FA + air + N_2_ + 50%RH	100	1000	6.82E‐08	65 000 h^−1^	25	100	9.82E‐02	209.1	[10a]
72	Mn_1‐_ *_x_* Ce*_x_* O_2_/palygorskite	Co‐precipitation method	100	300 ppm FA + air	300	100	2.04E‐08	20 000 h^−1^	152	1	7.36E‐04	114	[81]
73	Palygorskite/Cu‐Mn oxides	Impregnated method	100	1000 ppm FA + 20% O_2_ + N_2_	1000	100	6.82E‐08	32 500 h^−1^	NA	NA	NA	217	[82]
74	MnO_2_/cellulose	Impregnated method	5	100 ppm FA + 20%O_2_ + He	100	50	3.41E‐09	50 000 h^−1^	125	65	1.60E‐01	NA	[41a]
75	CuO/MnO_2_/GFS	Impregnated method	500	460 ppm FA + 21% O_2_ + N_2_	460	250	7.84E‐08	30 000 mL g^−1^ h^−1^	110	87	4.91E‐02	91	[83]
76	NiCo‐LDH/MnO_2_	Hydrothermal method	100	100 ppm FA + 21% O_2_ + N_2_	100	100	6.82E‐09	60 000 mL g^−1^ h^−1^	75	100	2.45E‐02	NA	[44b]
77	Co_3_O_4_/nickel foam	Hydrothermal method	3000	1000 ppm FA + 20% O_2_ + N_2_	1000	280	1.91E‐07	20 000 h^−1^	135	50	1.15E‐02	NA	[10b]
78	3D‐CeO_2_/CN	Precipitation method and calcination	60	90 ppm FA + 21% O_2_ + N_2_ + 50%RH	90	100	6.13E‐09	100 000 mL g^−1^ h^−1^	153	40	1.47E‐02	255.8	[10c]

NA: Not applicable

The TMO‐based catalyst systems showed high FA degradation rates at low temperature. For instance, the top five thermocatalysts performers, if sorted out in terms of r value, were ammonia modified *δ*‐MnO*_x_*/activated carbon (*δ*‐MnO*_x_*/AC‐N) (r: 2.39E‐02 mmol mg^−1^ h^−1^) > C@MnO (r: 1.47E‐02 mmol mg^−1^ h^−1^) > birnessite manganese oxide catalysts (r: 1.25E‐02 mmol mg^−1^ h^−1^) > carbon sphere/MnO_2_ (r: 4.91E‐03 mmol mg^−1^ h^−1^) > MnO_2_/PEG (r: 4.75E‐02 mmol mg^−1^ h^−1^)^[^ ^23^, ^43^, ^44^, ^61^ ^]^ (Table 1). FA catalytic degradation properties of TMO composite‐based catalysts outperformed the monometallic and bimetallic TMO‐based catalysts at low temperature (Table 1). The superior performance of TMO‐based composite catalysts (e.g., *δ*‐MnO*_x_*/AC‐N) is attributed to the following reasons. i) *δ*‐MnO*_x_*‐supported AC possessed a large content of oxygen vacancies for FA degradation. ii) The ammonia modification of *δ*‐MnO*_x_*/AC provided more basic sites for the adsorption of FA onto surface of *δ*‐MnO*_x_*/AC. iii) The surface oxygen vacancies (OVs) of *δ*‐MnO*_x_* can promote the adsorption and activation of O_2_ for forming active O, which makes FA oxidation more thermodynamically favorable.^[^ ^23b^ ^]^

The high degradation efficiency of TMO‐based composites over other performers was also confirmed when FA degradation performance was evaluated for different TMO‐based thermocatalysts at high reaction temperature (Table 2). The top five performers selected in terms of r values ranked in the order of MnO_2_/cellulose (r: 1.60E‐01 mmol mg^−1^ h^−1^) > graphene/MnO_2_ (r: 9.82E‐02 mmol mg^−1^ h^−1^) = alkali treated birnessite MnO_2_ (r: 9.82E‐02 mmol mg^−1^ h^−1^) > K modified MnO_2_ (r: 9.23E‐02 mmol mg^−1^ h^−1^) > 3D‐MnO_2_ (r: 7.36E‐02 mmol mg^−1^ h^−1^) > MnO*_x_*‐CeO_2_ (r: 7.12E‐02 mmol mg^−1^ h^−1^)^[^ ^10^, ^33^, ^36^, ^41^, ^54^, ^58^ ^]^ (Table 2). The superior performance of the MnO_2_/cellulose composite was mainly attributed to the synergistic effects of the abundant active sites provided by MnO_2_ and high FA adsorption ability of porous cellulose.^[^ ^41a^ ^]^ In addition, it was revealed that the FA catalytic degradation efficiency of high temperature TMO‐based thermocatalysts (e.g., MnO_2_/cellulose, r: 1.60E‐01 mmol mg^−1^ h^−1^) was approximately seven times larger than that of low temperature TMO‐based composites thermocatalysts (e.g., *δ*‐MnO*_x_*/AC‐N, r: 2.39E‐02 mmol mg^−1^ h^−1^).^[^ ^23^, ^41^ ^]^ Such results might suggest that the high temperature was beneficial for maximizing e^−^/h^+^ in the TMO‐based composite thermocatalysts to promote the FA degradation through the generation of reactive oxygen species (e.g.,^●^O_2_^−^). In light of the above comparison, the FA catalytic performance of TMO‐based composite catalysts outperformed the other types TMO‐based catalysts (e.g., mono‐TMO) in terms of r values for both low/high temperature conditions as defined above.

## 6 Conclusions and Outlook

The present review was organized to report the recent scientific developments of TMO‐based thermocatalysts used for the degradation of gaseous FA. TMO‐based catalysts have drawn great attention because of their availability, thermal stabilities, abundance, and cost‐effectiveness. The mechanisms of FA removal over the TMO‐based catalysts have also been discussed. To provide an in‐depth discussion on applied TMO‐based catalysts for thermal degradation of gaseous FA, monometallic, bimetallic, and composite‐based TMO thermocatalysts were compared. Accordingly, the FA catalytic performance of the TMO‐based catalysts was significantly affected by a number of factors (e.g., exposed crystal facets, alkali metal/nitrogen modification, alkali/acid treatment, and precursor type) that influence the generation of abundant TM cations, provide a high content of oxygen vacancies, activation of adsorbed O_2_, and enhancement of FA adsorption. Finally, the quantitative performance evaluation of diverse applied TMO‐based catalysts was carried out based on their kinetic reaction rate values. The FA catalytic performance of the TMO‐based catalysts was evaluated using kinetic reaction rate as the suitable metric under both low and high reaction temperature conditions. Based on this evaluation, TMO‐based composites catalysts outperformed other types of TMO‐based catalysts (i.e., mono‐TMO and bi‐TMO based catalysts) under all temperature conditions. As such, the TMO‐based composites catalysts appeared to be the more feasible option than mono‐ and bi‐TMO based catalysts for FA degradation. These observations may offer clear guidance for better design strategies for developing an effective thermocatalytic reactor for large scale applications.

Despite the great potential of TMO‐based thermocatalysts for FA removal, more efforts are needed to expand their real‐word applications as explained below.

1. It was observed that TMO composite thermocatalysts possessing functional matrices (e.g., cellulose and activated carbon) exhibited superior FA degradation performance compared to pristine TMO catalysts. Therefore, we recommend investigating the effect of other advanced materials like metal organic frameworks (e.g., large specific area and chemical stability) as matrices to further improve the catalytic performances of pure TMO‐based thermocatalysts.
2. An ample understanding of the interactions between pollutants and TMO‐based catalysts may help researchers develop efficient catalysts. For this purpose, the use of computational simulations such as density function theory and molecular dynamic simulations may have to be considered.
3. As most TMO‐based thermocatalysts are commonly prepared with a batch synthesis mode, they may suffer from some drawbacks (e.g., low efficiency, lack of flexibility, and tenability/controllability toward better product properties). For the upscaled application of TMO‐baed thermocatalysts, the novel synthesis routes are to be developed with the proper control of properties (e.g., size/shape and oxygen vacancies).
4. In most of the lab‐scale studies, thermocatalytic removal of FA on TMO‐based catalysts has been assessed under highly favorable reaction conditions (e.g., single pollutant system, high FA concentrations (>100 ppm), and large catalysts mass). However, FA is frequently present in the sub‐ppm or ppb level in the real indoor environment. To obtain more practical information for the removal of FA under real‐world conditions, the performances of TMO‐based catalysts should be evaluated properly to reflect the real‐world conditions (e.g., low concentration of FA and the presence of interfering pollutants).
5. Based on the performance evaluation, the TMO‐based composite catalysts are found to have superior degradation efficiencies at elevated temperature. Hence, in the pursuit of the cost‐effective TMO‐based catalysts, it is desirable to develop some strategies (e.g., alkali/acid treatment) for their practical operation under low temperature (e.g., room temperature) conditions.

## Conflict of Interest

The authors declare no conflict of interest.

## References

[1] a) K. Vikrant , K.‐H. Kim , E. E. Kwon , D. W. Boukhvalov , Chem. Eng. J. 2021, 433, 133497;

[2] a) T. Salthammer , S. Mentese , R. Marutzky , Chem. Rev. 2010, 110, 2536; 20067232 10.1021/cr800399g PMC2855181

[3] A. Yusuf , C. Snape , J. He , H. Xu , C. Liu , M. Zhao , G. Z. Chen , B. Tang , C. Wang , J. Wang , Catal. Rev. 2017, 59, 189.

[4] R. Li , Y. Huang , D. Zhu , W. Ho , S. Lee , J. Cao , Aerosol Sci. Eng. 2020, 4, 147.

[5] a) S. Zhu , J. Wang , L. Nie , ChemistrySelect 2019, 4, 12085;

[6] H. Guo , Z. Zhang , H. Hojo , M. Chen , H. Einaga , W. Shangguan , Catal. Surv. Asia 2019, 23, 199.

[7] a) C. Zhang , H. He , K.‐i. Tanaka , Catal. Commun. 2005, 6, 211;

[8] J. Quiroz , J.‐M. Giraudon , A. Gervasini , C. Dujardin , C. Lancelot , M. Trentesaux , J.‐F. Lamonier , ACS Catal. 2015, 5, 2260.

[9] H. Chen , Q. Wu , Y. Wang , Q. Zhao , X. Ai , Y. Shen , X. Zou , Chem. Commun. 2022, 58, 7730. 10.1039/d2cc02299k 35758107

[10] a) L. Shi , X. Zhou , Y. Guo , Y. Li , C. Yan , Q. Han , L. Zhang , W. Zhang , J. Hazard. Mater. 2023, 441, 129836; 36088878 10.1016/j.jhazmat.2022.129836

[11] P. Wu , X. Jin , Y. Qiu , D. Ye , Environ. Sci. Technol. 2021, 55, 4268. 33720707 10.1021/acs.est.0c08179

[12] a) K. Li , X. Luo , X. Lin , F. Qi , P. Wu , J. Mol. Catal. A: Chem. 2014, 1, 383;

[13] a) C. Ma , D. Wang , W. Xue , B. Dou , H. Wang , Z. Hao , Environ. Sci. Technol. 2011, 45, 3628; 21375237 10.1021/es104146v

[14] a) Z. Wang , W. Wang , L. Zhang , D. Jiang , Catal. Sci. Technol. 2016, 6, 3845;

[15] a) E. W. McFarland , H. Metiu , Chem. Rev. 2013, 113, 4391; 23350590 10.1021/cr300418s

[16] a) Y. Huang , B. Long , M. Tang , Z. Rui , M.‐S. Balogun , Y. Tong , H. Ji , Appl. Catal., B 2016, 181, 779;

[17] H. Zhang , Z. Zhang , Y. Liu , X. Fang , J. Xu , X. Wang , X. Xu , J. Phys. Chem. Lett. 2021, 12, 9188. 34528804 10.1021/acs.jpclett.1c02471

[18] L. Zhu , J. Wang , S. Rong , H. Wang , P. Zhang , Appl. Catal., B 2017, 211, 212.

[19] J. Zhang , Y. Li , L. Wang , C. Zhang , H. He , Catal. Sci. Technol. 2015, 5, 2305.

[20] Y. Chen , Y. Guo , H. Hu , S. Wang , Y. Lin , Y. Huang , Inorg. Chem. Commun. 2017, 82, 20.

[21] a) C. He , J. Cheng , X. Zhang , M. Douthwaite , S. Pattisson , Z. Hao , Chem. Rev. 2019, 119, 4471; 30811934 10.1021/acs.chemrev.8b00408

[22] N. Xiang , Y. Bai , Q. Li , X. Han , J. Zheng , Q. Zhao , Y. Hou , Z. Huang , Mol. Catal. 2022, 528, 112519.

[23] a) R. Li , Y. Huang , D. Zhu , W. Ho , J. Cao , S. Lee , Environ. Sci. Technol. 2021, 55, 4054; 33657800 10.1021/acs.est.1c00490

[24] a) S. Selvakumar , N. Nuns , M. Trentesaux , V. S. Batra , J. M. Giraudon , J. F. Lamonier , Appl. Catal., B 2018, 223, 192;

[25] a) K. Vikrant , S. Weon , K.‐H. Kim , M. Sillanpää , Appl. Mater. Today 2021, 23, 100993;

[26] a) L. Lu , H. Tian , J. He , Q. Yang , J. Phys. Chem. C 2016, 120, 23660;

[27] a) B.‐T. Teng , S.‐Y. Jiang , Z.‐X. Yang , M.‐F. Luo , Y.‐Z. Lan , Surf. Sci. 2010, 604, 68;

[28] a) S. Rong , P. Zhang , F. Liu , Y. Yang , ACS Catal. 2018, 8, 3435;

[29] a) F. Hashemzadeh , M. M. Kashani Motlagh , A. Maghsoudipour , J. Sol‐Gel Sci. Technol. 2009, 51, 169;

[30] R. Chen , Z. Sun , C. Hardacre , X. Tang , Z. Liu , Catal. Rev. 2022, 1.

[31] B. Chen , B. Wu , L. Yu , M. Crocker , C. Shi , ACS Catal. 2020, 10, 6176.

[32] a) Z. Chen , Z. Jiao , D. Pan , Z. Li , M. Wu , C.‐H. Shek , C. M. L. Wu , J. K. L. Lai , Chem. Rev. 2012, 112, 3833; 22462582 10.1021/cr2004508

[33] a) Z. Ye , Z. Jiajia , W. Guisheng , M. Dongsen , L. Guanzhong , Chem. J. Chin. Univ. 2014, 35, 2598;

[34] T. Chang , Z. Wang , H. An , F. Li , W. Xue , Y. Wang , J. Environ. Chem. Eng. 2022, 10, 108053.

[35] X. Yu , J. He , D. Wang , Y. Hu , H. Tian , Z. He , J. Phys. Chem. C 2012, 116, 851.

[36] a) X. Tang , Y. Li , X. Huang , Y. Xu , H. Zhu , J. Wang , W. Shen , Appl. Catal., B 2006, 62, 265;

[37] a) S. Lu , F. Wang , C. Chen , F. Huang , K. Li , J. Rare Earths 2017, 35, 867;

[38] Y. Huang , K. Ye , H. Li , W. Fan , F. Zhao , Y. Zhang , H. Ji , Nano Res. 2016, 9, 3881.

[39] a) C. Shi , Y. Wang , A. Zhu , B. Chen , C. Au , Catal. Commun. 2012, 28, 18;

[40] Z. Zhang , Z. Fan , H. Guo , W. Fang , M. Chen , W. Shangguan , Catal. Today 2019, 332, 139.

[41] a) L. Zhou , J. He , J. Zhang , Z. He , Y. Hu , C. Zhang , H. He , J. Phys. Chem. C 2011, 115, 16873;

[42] D. Sun , S. Wageh , A. A. Al‐Ghamdi , Y. Le , J. Yu , C. Jiang , Appl. Surf. Sci. 2019, 466, 301.

[43] C. Zhang , Y. Wang , W. Song , H. Zhang , X. Zhang , R. Li , C. Fan , J. Porous Mater. 2020, 27, 801.

[44] a) S.‐B. Do , S.‐E. Lee , T.‐O. Kim , Appl. Surf. Sci. 2022, 598, 153773;

[45] J. Li , P. Zhang , J. Wang , M. Wang , J. Phys. Chem. C 2016, 120, 24121.

[46] J. Miyawaki , G.‐H. Lee , J. Yeh , N. Shiratori , T. Shimohara , I. Mochida , S.‐H. Yoon , Catal. Today 2012, 185, 278.

[47] Y. Cao , H. Yu , F. Peng , H. Wang , ACS Catal. 2014, 4, 1617.

[48] a) S. L. Candelaria , Y. Shao , W. Zhou , X. Li , J. Xiao , J.‐G. Zhang , Y. Wang , J. Liu , J. Li , G. Cao , Nano Energy 2012, 1, 195;

[49] a) V. Soni , V. Goel , P. Singh , A. Garg , Int. J. Chem. React. Eng. 2021, 19, 1;

[50] a) J. Deng , W. Song , L. Chen , L. Wang , M. Jing , Y. Ren , Z. Zhao , J. Liu , Chem. Eng. J. 2019, 355, 540;

[51] J. E. Lee , Y. S. Ok , D. C. W. Tsang , J. Song , S.‐C. Jung , Y.‐K. Park , Sci. Total Environ. 2020, 719, 137405. 32114230 10.1016/j.scitotenv.2020.137405

[52] S. Wang , J. Xie , M. Wu , F. Wang , Appl. Surf. Sci. 2023, 156803.

[53] a) W. Wang , M. O. Tadé , Z. Shao , Prog. Mater. Sci. 2018, 92, 33;

[54] a) J. Wang , J. Li , P. Zhang , G. Zhang , Appl. Catal., B 2018, 224, 863;

[55] X. Chen , M. Chen , G. He , F. Wang , G. Xu , Y. Li , C. Zhang , H. He , J. Phys. Chem. C 2018, 122, 27331.

[56] a) Z. Fan , Z. Zhang , W. Fang , X. Yao , G. Zou , W. Shangguan , Chin. J. Catal. 2016, 37, 947;

[57] a) C. Zhang , Y. Li , Y. Wang , H. He , Environ. Sci. Technol. 2014, 48, 5816; 24738832 10.1021/es4056627

[58] J. Wang , G. Zhang , P. Zhang , J. Mater. Chem. A 2017, 5, 5719.

[59] a) J. Q. Torres , J.‐M. Giraudon , J.‐F. Lamonier , Catal. Today 2011, 176, 277;

[60] K. Vikrant , K.‐H. Kim , F. Dong , D. W. Boukhvalov , W. Choi , Chem. Eng. J. 2022, 428, 131177.

[61] H. Wang , Z. Huang , Z. Jiang , Z. Jiang , Y. Zhang , Z. Zhang , W. Shangguan , ACS Catal. 2018, 8, 3164.

[62] R. Fang , H. Huang , J. Ji , M. He , Q. Feng , Y. Zhan , D. Y. Leung , Chem. Eng. J. 2018, 334, 2050. 10.1016/j.chemosphere.2018.09.019 30223128

[63] Z. Dai , X. Yu , C. Huang , M. Li , J. Su , Y. Guo , H. Xu , Q. Ke , RSC Adv. 2016, 6, 97022.

[64] H. Xiaomei , C. Tianhu , W. Can , Z. Xuehua , H. Zhengyan , Acta Materiae Compositae Sin. 2022, 39, 1617.

[65] S. Rong , P. Zhang , J. Wang , F. Liu , Y. Yang , G. Yang , S. Liu , Chem. Eng. J. 2016, 306, 1172.

[66] Y. Zhang , Z. Zhao , D. Li , G. Cai , X. Tang , W. Li , D. Cheng , X. Wang , Cellulose 2022, 29, 7353.

[67] Z. Dai , J. Yu , Y. Si , Polymers 2022, 14, 2504. 35746080 10.3390/polym14122504 PMC9231320

[68] W. K. Zhao , J. Y. Zheng , C. B. Han , J. Ruan , Y. Lu , K. L. Zhou , T. R. Zhai , H. Wang , H. Yan , Chem. Eng. J. 2022, 440, 135877.

[69] J. Wang , J. Li , C. Jiang , P. Zhou , P. Zhang , J. Yu , Appl. Catal., B 2017, 204, 147.

[70] H. Tian , J. He , L. Liu , D. Wang , Z. Hao , C. Ma , Microporous Mesoporous Mater. 2012, 151, 397.

[71] L. Zhou , J. Zhang , J. He , Y. Hu , H. Tian , Mater. Res. Bull. 2011, 46, 1714.

[72] T. Chen , H. Dou , X. Li , X. Tang , J. Li , J. Hao , Microporous Mesoporous Mater. 2009, 122, 270.

[73] R. Wang , J. Li , Catal. Lett. 2009, 131, 500.

[74] H. Tian , J. He , L. Liu , D. Wang , Ceram. Int. 2013, 39, 315.

[75] H. Tian , J. He , X. Zhang , L. Zhou , D. Wang , Microporous Mesoporous Mater. 2011, 138, 118.

[76] Y. Wu , M. Ma , B. Zhang , Y. Gao , W. Lu , Y. Guo , RSC Adv. 2016, 6, 102127.

[77] L. Ma , D. Wang , J. Li , B. Bai , L. Fu , Y. Li , Appl. Catal., B 2014, 148–149, 36.

[78] H. Wang , W. Guo , Z. Jiang , R. Yang , Z. Jiang , Y. Pan , W. Shangguan , J. Catal. 2018, 361, 370.

[79] L. Xuesong , L. Jiqing , Q. Kun , W. HUANG , L. Mengfei , J. Rare Earths 2009, 27, 418.

[80] Y. Wen , X. Tang , J. Li , J. Hao , L. Wei , X. Tang , Catal. Commun. 2009, 10, 1157.

[81] C. Wang , H. Liu , T. Chen , C. Qing , X. Zou , J. Xie , X. Zhang , Appl. Clay Sci. 2018, 159, 50.

[82] P. Liu , G. Wei , H. He , X. Liang , H. Chen , Y. Xi , J. Zhu , Appl. Surf. Sci. 2019, 464, 287.

[83] W.‐J. Qiang , Q. Huang , J.‐H. Shen , Q.‐F. Ke , J.‐Y. Lü , Y.‐P. Guo , J. Cleaner Prod. 2022, 368, 133089.

